# A Case Report of Hydropic Gallbladder Presenting as Right Lower Quadrant Abdominal Pain

**DOI:** 10.21980/J8DD26

**Published:** 2025-04-30

**Authors:** Savannah Tan, Zoe Adams, Scott Rudkin, Danielle Matonis

**Affiliations:** * University of California, Irvine Medical Center, Department of Emergency Medicine, Orange, CA; ^ University of California, Irvine, School of Medicine, Irvine, CA

## Abstract

An 18-year-old female presented to the emergency department (ED) with two days of right lower quadrant pain and associated nausea and emesis. After relevant information was gathered and with physical exam findings of a tender right lower quadrant, positive psoas sign, positive Rovsing sign, and pain with right heel tap, the patient was presumed to have appendicitis. However, imaging contradicted the initial leading diagnosis and revealed a markedly distended, hydropic gallbladder with its tip near the umbilicus. Findings of the distended gallbladder with marked wall thickening and pericholecystic fat stranding and edema confirmed acute cholecystitis, and the patient was taken by general surgery for cholecystectomy. Together, this unusual presentation and this unexpected diagnosis shine light upon another facet of the hydropic gallbladder while also serving as a salient reminder to contemplate a broad differential regardless of seemingly classic presentations of illnesses.

**Topics:**

Cholecystitis, hydropic gallbladder, abdominal pain, appendicitis.

**Figure:**
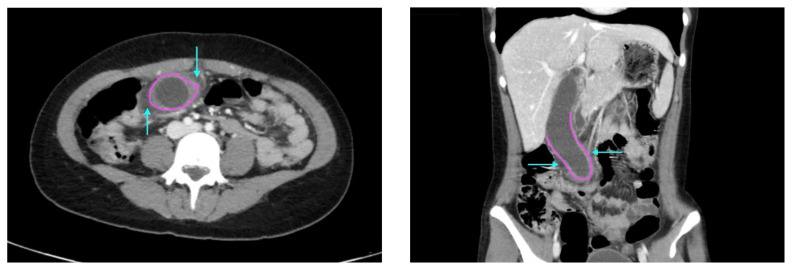
Video Link: https://youtube.com/shorts/97_wO65jh0M

## Brief introduction

Gallbladder disease is estimated to affect 6.3 million men and 14.2 million women between the ages of 20 and 74 in the United States alone.^1^ Of the forms of gallbladder disease, a rarer yet pertinent subcategory is hydropic gallbladder, a condition in which colorless, mucous-like fluid builds up in the gallbladder, replacing bile salts that are gradually reabsorbed.

While cholecystitis is typically caused by obstruction, the hydropic gallbladder is rather suspected to be caused by increased secretions from gallbladder mucosa, leading to gallbladder distention even without calculi or inflammation, distinct from acalculous cholecystitis.^2^ While hydropic gallbladder is known to symptomatically present in a fashion similar to acute cholecystitis, this case presents an atypical account in which the clinical presentation prior to radiological imaging aligned with acute appendicitis.

## Presenting concerns and clinical findings

An 18-year-old female with no past medical history presented to the ED for two days of sharp right lower quadrant (RLQ) abdominal pain aggravated by walking with associated nausea and vomiting. The pain began in her right upper quadrant, then migrated to the RLQ. The pain was rated as an 8/10 in severity. She reports a loss of appetite and inability to eat food due to her symptoms. She had about four to five episodes of non-bloody vomiting per day. Vital signs were notable for tachycardia to 137 beats per minute (bpm) and a temperature of 37.6°C. Pertinent physical exam findings included a soft abdomen that was tender to palpation in the RLQ with rebound tenderness, positive psoas and Rovsing signs, negative obturator sign, and pain elicited with heel tap. Tenderness was not elicited on palpation of the right upper quadrant, with negative Murphy’s sign.

## Significant findings

Laboratory results were significant for leukocytosis to 18.1 × 10^9^ white blood cells/L (reference range: 4.5–11.0 x 10^9^ white blood cells/L) without leukocyte left shift. Calculation of the Alvarado score for acute appendicitis yielded a score of 9, indicating “very probable appendicitis.” The score was calculated as such: right lower quadrant tenderness (+2), elevated temperature greater than 37.3°C (+1), rebound tenderness (+1), migration of pain to the right lower quadrant (+1), anorexia (+1), nausea or vomiting (+1), and leukocytosis > 10,000 (+2). Computed tomography (CT) of the abdomen and pelvis with contrast was ordered, and general surgery was consulted for the initial working diagnosis of acute appendicitis. However, the CT scan resulted with findings of a markedly distended gallbladder measuring approximately 14.5 x 4 centimeters (cm) with marked gallbladder wall thickening (magenta) and pericholecystic fat stranding (cyan). The appendix was not dilated and had no inflammatory changes or edema. Follow-up right upper quadrant ultrasound confirmed the diagnosis of acute cholecystitis.

## Patient course

The final diagnosis of acute cholecystitis with a severely dilated hydropic gallbladder was made, and the patient was taken to the operating room for a laparoscopic cholecystectomy. She tolerated the procedure well without complications and was discharged home after a short recovery.

## Discussion

Acute cholecystitis is most commonly caused by gallstones, termed acute calculous cholecystitis, though acalculous cholecystitis accounts for 5–10% of cases.^3^ Physical examination findings of patients with acute cholecystitis include fever, tachycardia, RUQ or epigastric pain, guarding, and a positive Murphy’s sign. Sensitivity and specificity of a positive Murphy’s sign is 97% and 48%, respectively.^4^ Diagnosis of acute cholecystitis requires evidence of gallbladder wall thickening, pericholecystic fluid, or edema on abdominal ultrasound, sonographic Murphy’s sign, or failure of the gallbladder to fill during cholescintigraphy if ultrasound is equivocal.

Relatedly, gallbladder hydrops is another complication of cystic duct obstruction, also often caused by gallstones. While this condition goes by several other names, including gallbladder mucocele, gallbladder hydrocele, and/or hydropic gallbladder, it is ultimately diagnosed when mucous, mucous-like fluid, or water have replaced the brown or green bile normally present in the gallbladder.^5^ Over time, the gallbladder expands with these new clear fluids, creating pressure that leads to clinical signs similar to those seen in acute cholecystitis as mentioned above (i.e., fever, tachycardia, RUQ pain, etc.).

In contrast, acute appendicitis refers to inflammation of the appendix, which is located at the base of the cecum, most often caused by obstruction due to fecaliths, calculi, lymphoid hyperplasia, infectious processes, and benign or malignant tumors.^6^ Common physical examination manifestations of appendicitis include fever, periumbilical abdominal pain that eventually localizes to the RLQ, and positive psoas, obturator, or Rovsing’s signs. The Alvarado score is often used to stratify patients with these symptoms, typically with a cut-point score of 7 to “rule in” appendicitis and progression to surgery. However, this cut-off has an overall specificity of 81% and has been shown to be over-predicting the probability of acute appendicitis in women.^7^ Ultimate diagnosis of acute appendicitis is evaluated with CT of the abdomen and pelvis with intravenous contrast showing appendix wall thickening, enlarged appendiceal diameter, periappendiceal fat stranding, and wall enhancement. CT of the abdomen and pelvis has a sensitivity of 100% and specificity of 97% for diagnosing appendicitis.^8^

This patient had typical findings of acute appendicitis including the characteristic RLQ pain, anorexia, nausea and vomiting, and physical exam findings consistent with appendicitis including positive psoas and Rovsing signs and pain with heel tap. Despite high clinical suspicion, imaging contradicted the diagnosis of appendicitis, and instead revealed findings consistent with acute cholecystitis. While there are many factors and findings that are used to diagnose common conditions like appendicitis, it is important to remember they ultimately do not guarantee the disease. For example, despite tenderness at McBurney’s point being quintessential in the diagnosis of appendicitis, this finding has a sensitivity of 50–94% and a specificity of 75–86%.^9^ As a whole, this patient’s case serves not only as a helpful characterization of a relatively rare subset of gallbladder disease, but it also acts as a reminder to emergency medicine providers to consider a wide differential even in the face of classic presentations of common illnesses.

## Supplementary Information










