# Supplementary figures and images for: A Case Report of Hydropic Gallbladder Presenting as Right Lower Quadrant Abdominal Pain
Source: J Educ Teach Emerg Med. 2025 Apr 30;10(2):V14–6. doi: 10.21980/J8DD26 (PMC12054074; doi:10.21980/J8DD26)

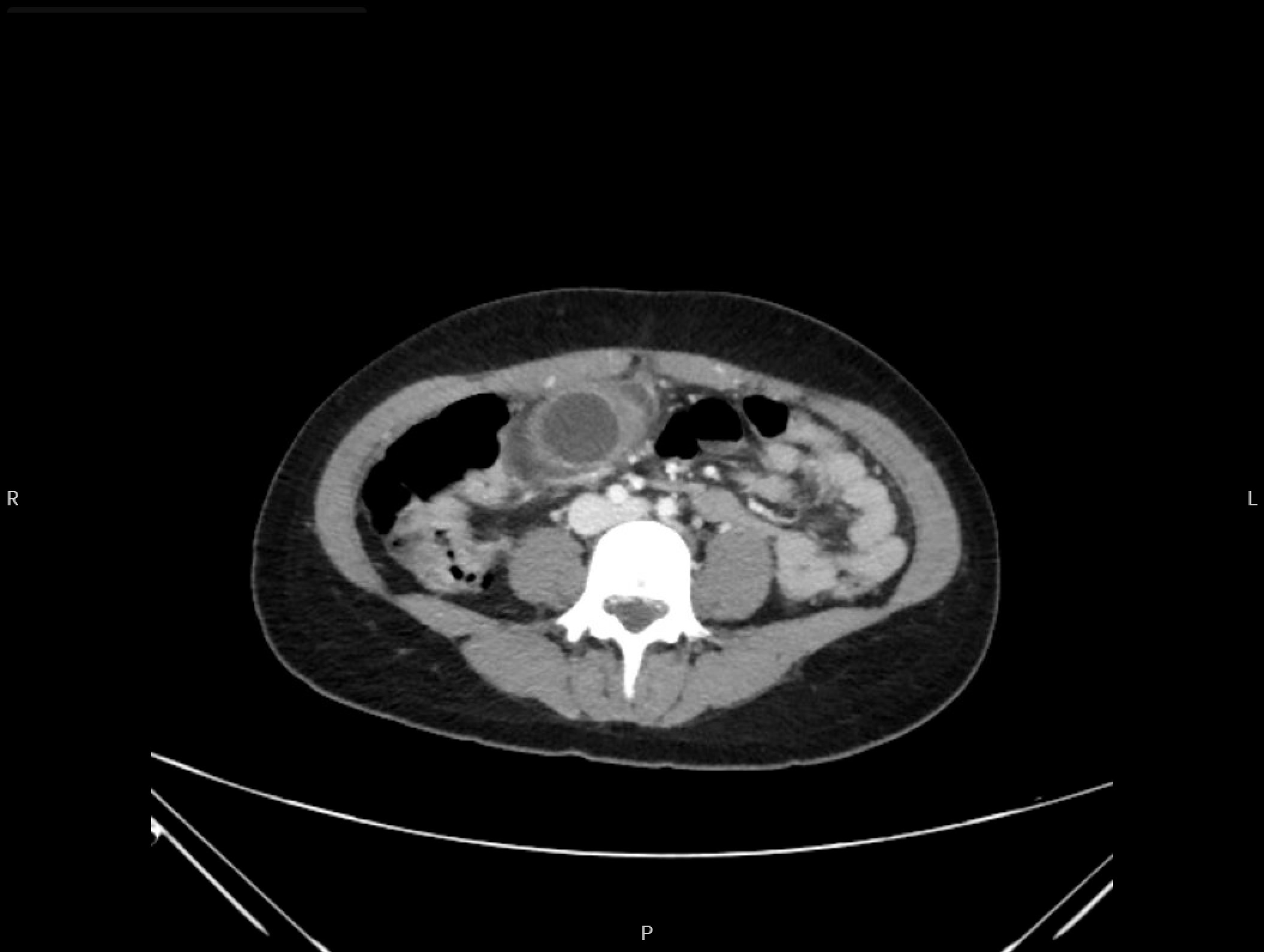

Supplement: Supplementary file 1 [file 10-2-V14-supp1.jpeg]

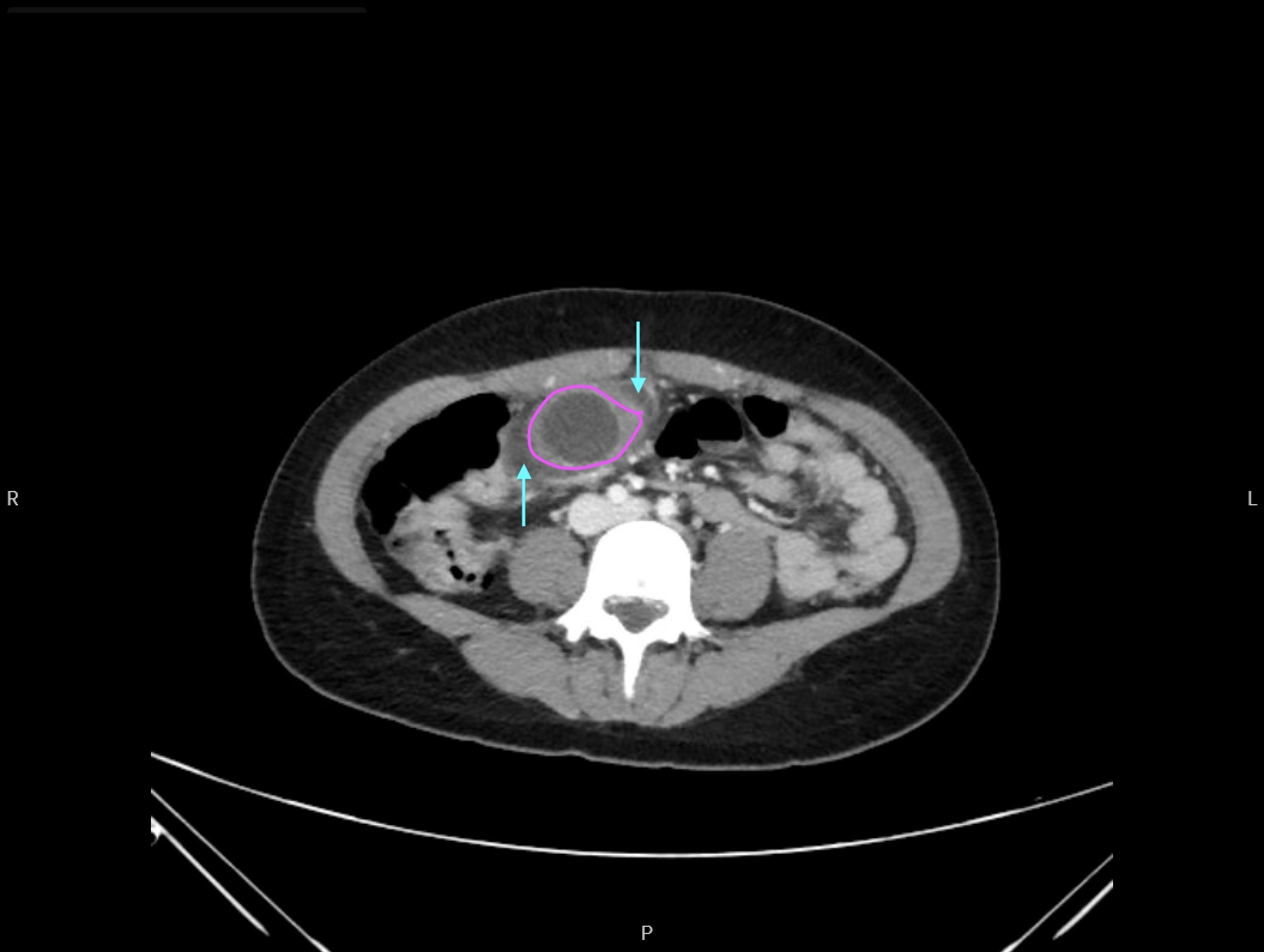

Supplement: Supplementary file 2 [file 10-2-V14-supp2.jpeg]

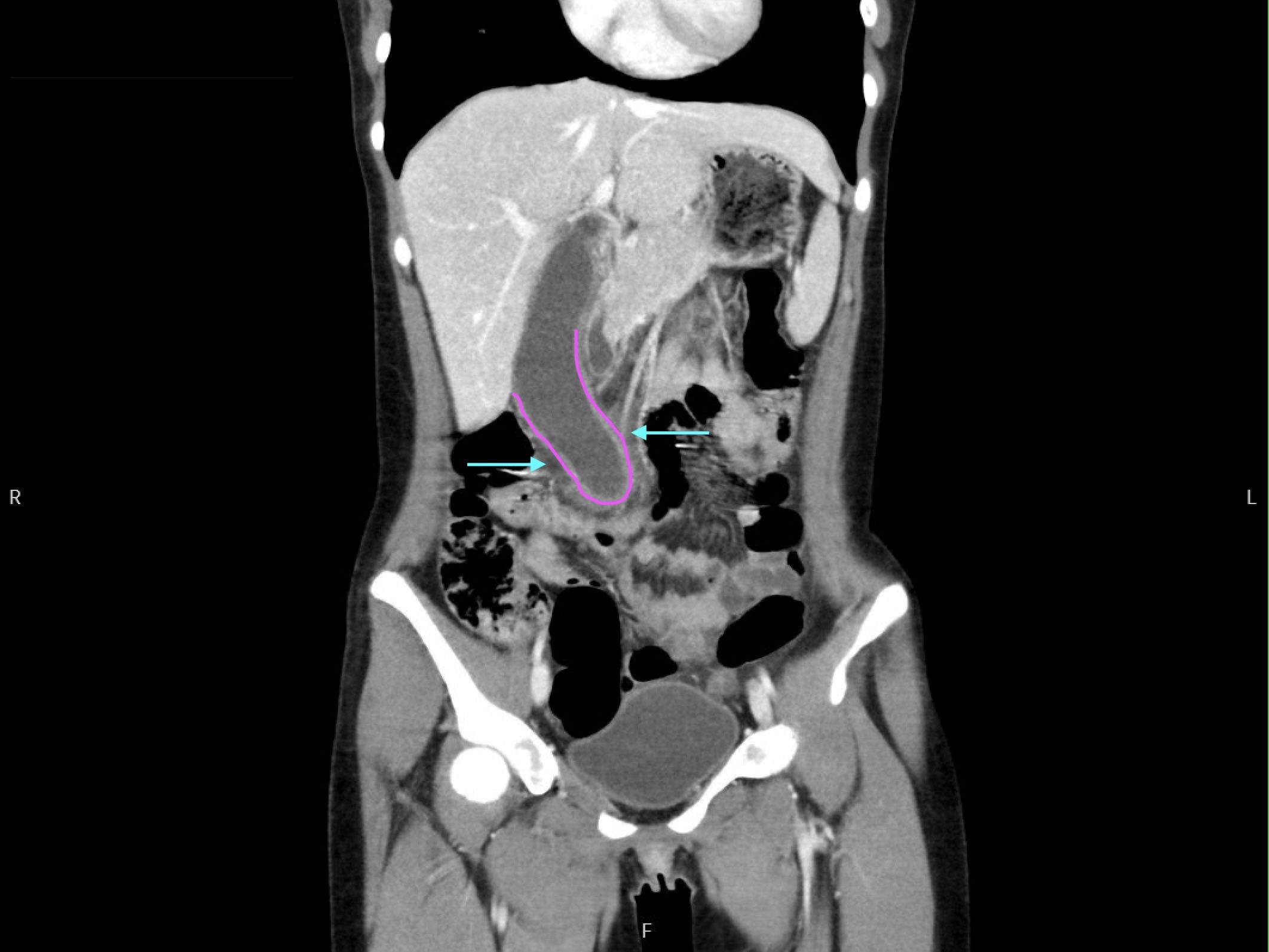

Supplement: Supplementary file 3 [file 10-2-V14-supp3.jpeg]

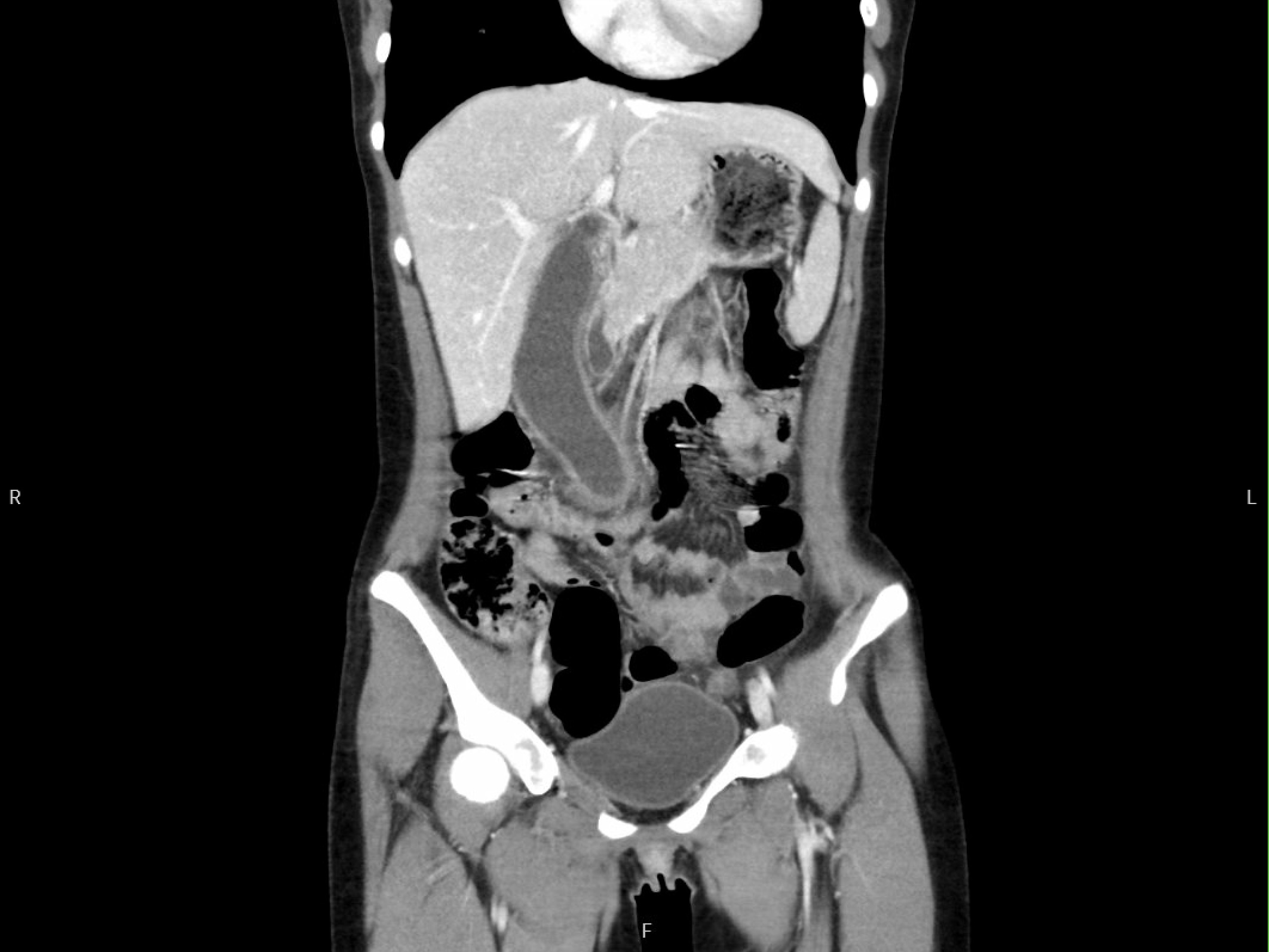

Supplement: Supplementary file 4 [file 10-2-V14-supp4.jpeg]
